# Short-term gut microbiota’s shift after laparoscopic Roux-en-Y vs one anastomosis gastric bypass: results of a multicenter randomized control trial

**DOI:** 10.1007/s00464-024-11154-6

**Published:** 2024-09-18

**Authors:** Flavio De Maio, Cristian Eugeniu Boru, Nunzio Velotti, Danila Capoccia, Giulia Santarelli, Ornella Verrastro, Delia Mercedes Bianco, Brunella Capaldo, Maurizio Sanguinetti, Mario Musella, Marco Raffaelli, Frida Leonetti, Giovani Delogu, Gianfranco Silecchia

**Affiliations:** 1 Dipartimento Di Scienze Di Laboratorio E Infettivologiche, Fondazione Policlinico A. Gemelli IRCCS, Università Cattolica del Sacro Cuore, Rome, Italy; 2 General Surgery Division, Sant’Andrea Hospital, Department of Medical Surgical Sciences and Translational Medicine and Department of Medical-Surgical Sciences and Biotechnologies, University “La Sapienza” of Rome, Via Di Grottarossa N. 1035, 00189 Rome, Italy; 3 Department of Advanced Biomedical Sciences, University of Naples “Federico II”, Naples, Italy; 4 Dipartimento Di Scienze Biotecnologiche Di Base, Cliniche Intensivologiche e Perioperatorie – Sezione di Microbiologia, Università Cattolica del Sacro Cuore, Rome, Italy; 5 Division of Endocrine and Metabolic Surgery, Fondazione Policlinico A. Gemelli IRCCS, Università Cattolica del Sacro Cuore, Rome, Italy; 6 Department of Clinical Medicine and Surgery, University of Naples ‘‘Federico II”, Naples, Italy; 7 Mater Olbia Hospital, Olbia, Italy

**Keywords:** Morbid obesity, One anastomosis gastric bypass, Roux-en-Y gastric bypass, Gut microbiota, Microbial diversity, Bariatric/metabolic surgery

## Abstract

**Background:**

Roux-en-Y (RYGB) and one anastomosis gastric bypass (OAGB) represent two of the most used bariatric/metabolic surgery (BMS) procedures. Gut microbiota (GM) shift after bypass surgeries, currently understated, may be a possible key driver for the short- and long-term outcomes.

**Methods:**

Prospective, multicenter study enrolling patients with severe obesity, randomized between OAGB or RYGB. Fecal and blood samples were collected, pre- (T0) and 24 months postoperatively (T1). GM was determined by V3-V4 16S rRNA regions sequencing and home-made bioinformatic pipeline based on Qiime2 plugin and R packages.

**Objects:**

To compare OAGB *vs* RYGB microbiota profile at T1 and its impact on metabolic and nutritional status.

**Results:**

54 patients completed the study, 27 for each procedure. An overall significant variation was detected in anthropometric and serum nutritional parameters at T1, with a significant, similar decrease in overall microbial alpha and beta diversity observed in both groups. An increase in relative abundances of Actinobacteria and Proteobacteria and a reduction of Bacteroidetes, no significant changes in Firmicutes and Verrucomicrobia, with an increase of the Firmicutes/Bacteroidetes ratio were observed.

**Conclusions:**

BMS promotes a dramatic change in GM composition. This is the first multicenter, RCT evaluating the impact of OAGB vs Roux-en-Y bypass on GM profile. The bypass technique per se did not impact differently on GM or other examined metabolic parameters. The emergence of slightly different GM profile postoperatively may be related to clinical conditions or may influence medium or long-term outcomes and as such GM profile may represent a biomarker for bariatric surgery’s outcomes.

**Graphical abstract:**

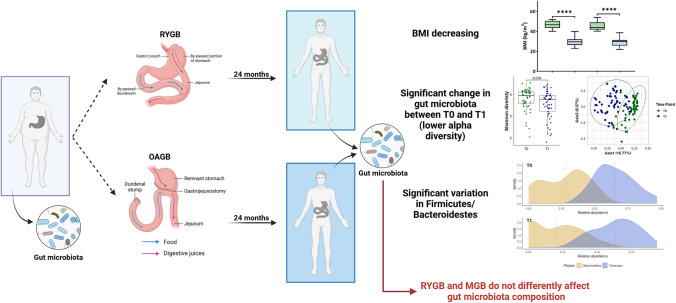

**Supplementary Information:**

The online version contains supplementary material available at 10.1007/s00464-024-11154-6.

The prevalence of obesity and its related comorbidities, including type 2 diabetes (T2D), are still rising worldwide [1]. Obesity is a complex and multifactorial disease, intertwining host biology, genetics, and environmental factors [2]. The gut microbiota (GM) has been described as a key contributor to both obesity and T2D, as changes of its composition, richness and functionality are associated with metabolic alterations, such as insulin resistance, low-grade systemic inflammation, and adiposity [3]. The adult gut bacterial community is characterized mainly by taxa belonging to two phyla, Firmicutes and Bacteroidetes whose relative proportions differ between populations, with obese subjects showing reduced *Bacteroidetes* and increased *Firmicutes* relative proportions [4–6]. In fact, it has been proposed that GM may contribute to obesity by altering the proportions in these and other critical bacterial phyla [6–8].

Minimally invasive bariatric/metabolic surgery (BMS) remains the best option proficient to induce long-lasting weight loss [9], alongside significant improvements of all obesity-associated comorbidities [6, 10]. Laparoscopic Roux-en-Y gastric bypass (RYGB) and One Anastomosis Gastric Bypass (OAGB) are currently the two most common bypass bariatric procedures used [11, 12]. The OAGB has been improved during the last 20 years [13, 14], evolving as an effective, standard, and safe procedure with only one omega-loop gastro-jejunal anastomosis [15], endorsed by the International Federation Surgical Obesity (IFSO) [16]. OAGB provides similar results at mid-term as concern weight loss and control/remission of metabolic comorbidities as RYGB [17], though some controversies remain as biliary reflux and potential increased risk of esophageal cancer [18–20].

Among the mechanisms involved in post-bariatric surgery metabolic improvements and weight loss, modulations of the composition and functionality of GM have been postulated [7, 8]. GM may mediate some of the metabolic effects of BMS, and changes in its composition and diversity have been observed in the short and long-term after RYGB in humans [6, 21]. Recent findings point to a direct role for the GM in mediating improved metabolic health post-RYGB surgery [22]. However, there are no prospective investigations of these changes after OAGB vs RYGB in a homogenous cohort of patients with severe obesity.

## Materials and methods

### Study design, patient enrollment, examination, and surgical approach

This is a multicenter, prospective, randomized study, enrolling 84 patients with severe obesity, candidates to laparoscopic BMS between May 2018 and January 2020 in three Italian, academic, high-volume centers. Inclusion and exclusion criteria, study’s characteristics and design, approvals, and registration on clinicaltrial.gov (Unique Protocol ID: NCT03412149) were previously reported [23]. Individuals meeting BMS eligibility requirements as aged 18–65 years old, with a body mass index (BMI) 40–55 kg/m^2^, non-smokers, candidates for primary laparoscopic OAGB or RYGB, without any concomitant surgeries except hiatal hernia repair. The study was conducted in accordance with the principles of CONSORT 2010 statement [24]. The protocol did not change during the study’s period. Exclusion criteria were corticosteroids use, vitamin E, fish oil treatment; antibiotics or probiotics treatment 2 months prior to surgery; chronic gastrointestinal diseases or syndromes; previous bariatric and/or hepato-biliary-pancreatic surgery; and gallstones. All patients received and signed a specific consent, related to the bariatric operations, approved by the national society, and to the participation in the study.

Before undergoing surgery (T0) patients underwent physical examination, fasting blood tests for metabolic and nutritional parameters assessment, including T2D and dyslipidemia, and multidisciplinary team evaluation following recommendation of Italian and international guidelines [16, 23]. A standardized meal test was performed at T0 and T1, with 250 ml of liquid meal Oxepa® (Abbott, Tokyo, Japan 375 cal, 55.5% fat, 28% carbohydrate, 16.5% protein). Blood samples were collected at baseline, 30, 60, 90 and 120 min after the meal test to assess the concentration of circulating lipids and stored at –80 °C. We analyzed microbiota at T0 and T1 before the meal test, so that meal test did not impact on microbiota.

The evidence of severe esophagitis (Los Angeles classification ≥ B), severe gastroesophageal reflux disease GERD and large hiatal hernia were considered as exclusion criteria.

BMS was indicated following national and international guidelines [4, 25]. Patients received OAGB or RYGB based on a computerized random choice (randomization 1:1, random.org), with no restriction, performed by the coordinating center. Patients and physicians allocated to the intervention group were aware of the allocated arm in the surgery day, while outcome assessors and data analysts were kept blinded to the allocation.

Briefly, the surgical technique of double loop RYGB, approved and reproduced by all participants consists in the making of a 5–6 cm long gastric pouch (measured from the gastroesophageal junction to obtain a volume of 30–40 ml) using three or four 45 mm linear staplers. An antecolic, antegastric 75 cm small bowel loop brought up and anastomosed to the gastric pouch, performing a 3 cm gastro-jejunostomy by linear stapler followed by manual closure of the gastroenterotomy with 3/0 absorbable barbed suture. A 150 cm alimentary limb is afterwards created, with latero-lateral, entero-enteral linear stapled anastomosis, while the enterotomy is closed in two layers with 3/0 absorbable barbed suture. Finally, the omega loop between the two anastomoses was divided, to create the R-en-Y.

The OAGB technique approved and reproduced by all participants consists in the making of a narrow gastric pouch on the lesser curve (longer then 12 cm measured from the gastroesophageal junction), starting below the incisura angularis with a transverse resection of 4–5 cm, using four or five 60 mm linear staplers. An antecolic, antegastric 160–200 cm small bowel loop (measured from the Treitz ligament) was brought up and anastomosed to the gastric pouch using one linear stapler, followed by manual closure of the gastroenterotomy with 3/0 absorbable barbed suture.

The lengths of limbs were carefully measured (stretch) with a graduated instrument. The mesenteric defects were closed in case of RYGB. In the case of enlarged hiatal defect, concomitant posterior cruroplasty was performed with two or three interrupted non absorbable sutures, after proper dissection of the hiatal area and abdominalization of the distal esophagus for at least 4–5 cm, calibrated on a 42-Fr bougie.

A standard, supplementary regimen was recommended to all operated patients, including vitamin D and iron. All patients repeated a complete work-up 24 months postoperatively (T1), including upper gastrointestinal endoscopy (EGD).

### Sample collection and DNA isolation

**Fig. 1:**
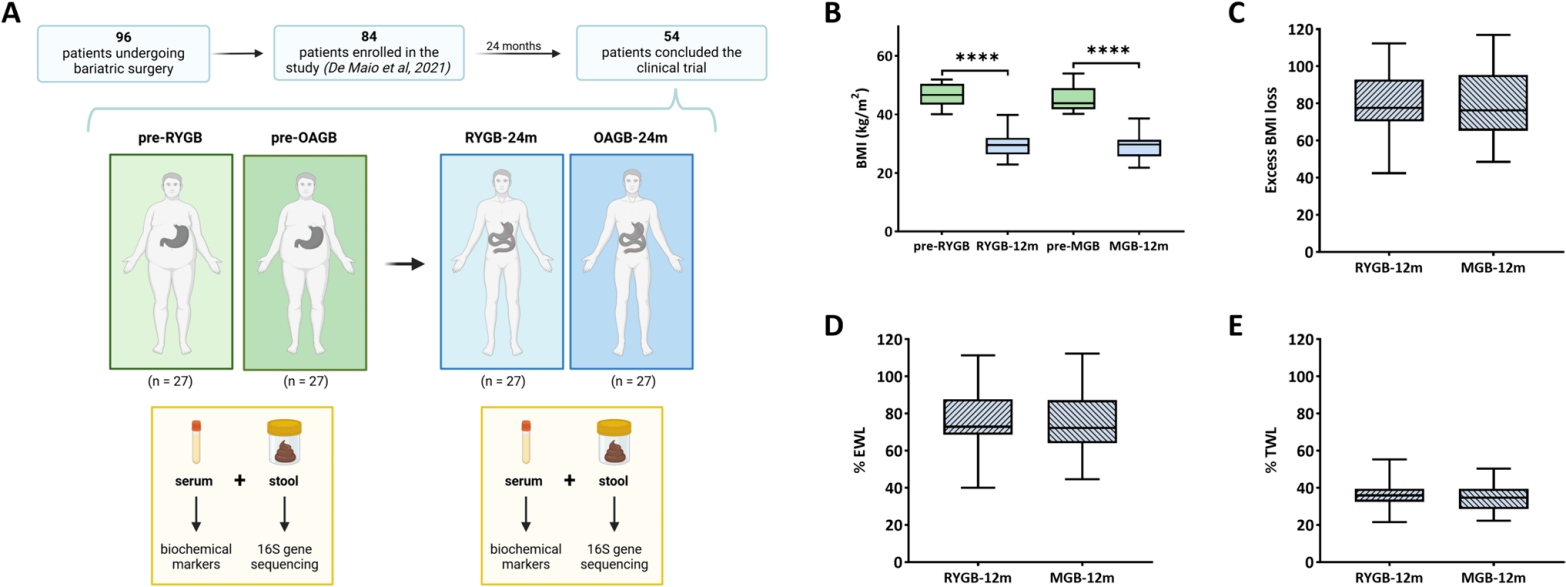
Schematic representation of the study’s design and timeline, and weight-based measures before bariatric surgery and 24 months postoperatively. Gut microbiota and serum biochemical parameters of a 54 patients cohort undergoing bariatric surgery, Roux-en-Y Gastric Bypass (RYGB) and One Anastomosis/Mini Gastric Bypass (OAGB/MGB), were examined before surgical intervention (T0, 84 patients enrolled) and 24 months later (T1, 54 patients that completed the follow-up) **A** Body Mass Index (BMI) **B**, excess BMI **C**, the percentage of excess weight loss (%EWL) and the percentage of total weight loss (%*TWL) were measured *(**D**, **E**)

Fresh stool samples were collected before and after surgical intervention from the 54 patients included in the study (Fig. 1a). All samples were stored at −80 °C until processing. Sampling was performed according to the standard protocols and regulation, including patients who had not taken antibiotics or probiotics in the previous 3 months.

DNA isolation was performed within a biological safety and sterile cabinet by using DANAGENE MICROBIOME Fecal DNA kit (DanaGen-Bioted, S.L.) following manufacturer’s instructions [26]. Briefly, microbial DNA was isolated from ≈200 mg of fecal samples homogenized in Cationic detergent cetyltrimethylammonium bromide (CTAB) buffer. The extracted DNA was stored at −20 °C until further analysis. Before library preparation, the isolated DNA was quantitatively evaluated by using Qubit 4.0 fluorometer (Life Technologies) and the Qubit dsDNA HS (High Sensitivity) assay kit (Life Technologies).

V3–V4 hypervariable regions from the 16S rRNA gene were amplified by using the following primers: V3_Next_For, 5’-TCGTCGGCAGCGTCAGATGTGTATAAGAGACAGCCTACGGGNGGCWGCAG-3’ and V4_Next_Rev, 5’-TCTCGTGGGCTCGGAGATGTGTATAAGAGACAGGACTACHVGGGTATCTAATCC-3’, which were designed to contain (from 5’ to 3’), the sequences for the Nextera transposon and for BV5 (Next For) and AV6 (Next Rev) priming [23, 27]. Extracted DNA (2.0 ng) was used as the template in a 50-µL PCR volume, which contained 1U Phusion High-Fidelity DNA polymerase (Thermo Fisher), 1X High-Fidelity buffer (Thermo Fisher), 200 μM dNTPs, and 0.3-μM each primer. Thermal cycling conditions were set as follows: (i) 98 °C for 30 s; (ii) 20 cycles, each consisting of 98 °C for 10 s, 55 °C for 30 s, and 72 °C for 15 s; (iii) 15 cycles, each consisting of 98 °C for 10 s, 62 °C for 30 s, and 72 °C for 15 s; and (iv) 72 °C for 7 min. Amplicons were purified using Agencourt AMPure XP beads (Beckman Coulter) and were eluted in 35-µL nuclease-free water. Amplicons were then checked for quality on 1% agarose gel electrophoresis (Thermo Fisher), and DNA concentration was determined using the above-mentioned method. To incorporate unique Nextera XT i5 and i7 indexes to both amplicon ends, we used 40 ng of purified amplicons as the template in a 50-µL PCR volume, which contained 1U Phusion High-Fidelity DNA polymerase, 1X High-Fidelity buffer, 100-μM dNTPs, and 5-μL each of i5 and i7 indexes. Thermal cycling conditions were set as follows: (i) 98 °C for 30 s; (ii) 5 cycles, each cycle consisting of 98 °C for 10 s, 63 °C for 30 s, and 72 °C for 3 min. Indexed amplicons were purified using Agencourt AMPure XP beads and eluted in 25 µL nuclease-free water, and amplicon quality and concentration was assessed. Each sample’s indexed amplicons were equimolarly diluted, and the final pool was subjected to 2 × 250 paired-end sequencing (Illumina) onto an Illumina MiSeq instrument. To increase the base-diversity degree, an internal control (PhiX v3; Illumina) was added to the DNA library.

### Bioinformatics and statistical analysis

Raw sequencing data were processed with QIIME2 (version 2020.6) in a home-made pipeline above described [23, 28]. Demultiplexing and quality inspection of paired-end reads were performed using the “demux” plugin, while trimming of Illumina adapter sequence (5’-CTGTCTCTTATACACATCT-3’) was performed using the “cutadapt trim-paired” plugin. Denoising of paired-end reads was performed using the “dada2 denoised-paired” plugin, which allowed to adjust the number of 5’- and 3’-end trimmed bases to remove primer sequences or low-quality sequences [29, 30]. This led to an approximately 70% good-merged reads output. Amplicon sequence variants (ASVs) were assembled using the “feature-table summarize” plugin, while we applied the “feature-classifier” plugin to classify ASVs at the taxonomic level by the VSEARCH global consensus alignment and the SILVA 132 16S rRNA database (at a 99% sequence similarity threshold). Sequences, ASVs, taxonomy, and metadata tables are available upon request to the corresponding author.

Data analysis was performed using R v4.0.2 (https://www.rstudio.com/) and the phyloseq software package [31]. First, ASVs for which a bacterial taxonomic assignment could not be achieved (i.e., unassigned ASVs) were removed and a couple of two samples were discharged because one sample showed less than 1000 reads. Finally, bacterial taxa that were not seen more than two times in at least 5% of the samples were removed. At the end of this process, 8,160,105 reads (median value = 78,519 reads) were obtained accounting for a total of 907 bacterial taxa. An additional taxonomic filter was applied to remove low-prevalence taxa (Patescibacteria and Tenericutes), generating a final dataset that accounted 8,159,323 reads (median value = 78,519reads) accounting for 904 taxa. To minimize the effect of sequencing depth variation across samples normalization to reads median reads was performed.

To measure alpha diversity, we used the final dataset to calculate the Richness (observed species), the Shannon index, Pielou’s evenness and Faith’s diversity (phylogenetic diversity). Statistical significance was assessed by using Wilcoxon-Mann–Whitney test or Kruskal–Wallis test.

To measure beta diversity, we used the final dataset to generate Weighted Unifrac and Bray Curtis distance matrices that were then represented by principal coordinate analysis (PCoA) [32]. Statistical significance was assessed using the vegan package’s adonis function, which performs permutational multivariate analysis of variance (PERMANOVA).

Relative abundances were calculated at Phylum and Genus taxonomic level and Wilcoxon-Mann–Whitney test or Kruskal–Wallis test were used for statistical significance assessment.

Differences in clinical characteristics across samples previously stratified were assessed using the Wilcoxon-Mann–Whitney test or Kruskal–Wallis -test for continuous variables or the Chi-square test for categorical variables. Results of biochemical parameters (serum glucose and vitamin D) were correlated to the relative abundances of the main phyla by Spearman’s rank correlation coefficient. Data distribution was evaluated by Shapiro–Wilk test before Spearman’s correlations. In all analyses, statistical significance was set at a *p* value of < 0.05.

## Results

### Main outcomes of bariatric surgery

No differences were found at T0 regarding anthropometric, nutritional parameters, except for vitamin D. The alpha and beta diversity examinations at T0 showed no statistical differences in GM profile between the enrolled patients [23]. As depicted in Fig. 1a, a total of 54 patients fulfilled all the inclusion criteria and completed a minimum 24-month follow-up, 27 for each surgical group. The original protocol previewed a main milestone at the end of 2020, one year after the last enrolled patients. Due to the Covid restrictions (2020 and 2021) and the difficulties in fecal and blood analyses obtainment, the final milestone for samples obtention was the end of 2021. This allowed a total of approximately 24 months minimum follow-up period, but with the price of almost 30% lost patients to follow-up. The *sample size* (*n* = 54, 27 for group) was calculated fixing alpha error, power, *effect size* (difference of Firmicutes Relative Abundance between the two groups) and standard deviation at 5%, 80%, 0.15 and 0.2, respectively.

Mortality, conversion rate to open surgery and hospital readmission for reoperation due to early or late surgical complications were nil in both groups. At T1 all patients were free of PPI treatment, undergone no other surgical procedures during the study period, followed a standard vitamin supplementation, according to international guidelines [32] and did not receive antibiotics at least one month before meal tests. Clinical characteristics at baseline T0 and at T1 are presented in Table 1. Two years after the surgery, a significant decrease in body weight, BMI, neck circumference, waist and hip was observed (*p* < 0.001), though no differences at T1 were observed between RYGB and OAGB (Table 1, Supplementary Table 1, Fig. 1B–E). Regarding metabolic and macronutrient parameters, hemoglobin, HbA1c, glucose, triglycerides, cholesterol, LDL, ferritin and cortisol were significantly reduced, while vitamin D was significantly increased.

**Table 1:** Anthropometric and biochemical parameters of the patients undergoing to Roux-en-Y Gastric Bypass (RYGB) and Mini Gastric Bypass (MGB) bariatric surgery. Participants were stratified following pre- and post-bariatric surgery as T0 and T1, respectively. Results of the Wilcoxon T-test for paired sample followed by Bonferroni’s correction were reported. Significant results (*p* < 0.05) are shown

	T0 median [Q1–Q3]	T1 median [Q1–Q3]	Wilcoxon *T*-test
Weight (kg)	128,5 [112,6**–**143,2]	81,00 [72,2**–**92,1]	p < 0.001
BMI (kg/m^2^)	44,8 [42,1**–**49,7]	29,6 [26,1**–**31,4]	p < 0.001
Neck circum (cm)	42,0 [38,0**–**46,0]	36,0 [34,0**–**39,0]	p < 0.001
Waist (cm)	127,5 [118,5**–**138,5]	95,5 [87,0**–**105,0]	p < 0.001
Hip (cm)	134,5 [128,0**–**140,0]	109,5 [100,0**–**116,0]	p < 0.001
Albumin (g/dL)	4,1 [3,9**–**4,4]	4,1 [3,9**–**4,3]	p = 0.846
Hemoglobin (g/dL)	13,6 [12,4**–**14,5]	13,2 [12,5**–**13,9]	p = 0.019
HbA1c (mmol/ml)	36,0 [33,0**–**41,5]	34,0 [30,0**–**37,0]	p < 0.001
Glucose (mg/dL)	92,0 [87,0**–**105.2]	83,00 [76,7**–**87,0]	p < 0.001
Triglycerides (mg/dL)	118,0 [95,0**–**149,2]	78,5 [64,5**–**103,5]	p < 0.001
Cholesterol (mg/dL)	191,6 [163,0**–**214,7]	154,00 [129,7**–**172,7]	p < 0.001
HDL (mg/dL)	48,0 [38,0**–**55,2]	52,5 [39,0**–**59,2]	p = 0.640
LDL (mg/dL)	122,5 [91,0**–**151,0]	92,0 [75,7**–**111,3]	p < 0.001
Ferritin (ng/mL)	63,0 [34,0**–**118,2]	42,4 [16,7**–**112,0]	p = 0.030
Iron (μg/dL)	64,9 [52,4**–**88,2]	75,5 [55,0**–**92,4]	p = 0.766
Calcium (mg/dL)	9,5 [9,1**–**9,7]	9,3 [9,0**–**9,6]	p = 0.120
Vitamin D (ng/mL)	20,9 [13,1**–**27,2]	28,3 [23,5**–**36,0]	p < 0.001
Vitamin B12 (pg/mL)	348,0 [247,0**–**418,0]	363,5 [286,5**–**510,5]	p = 0.207
Cortisol (μg/mL)	86,0 [19,8**–**114,0]	13,0 [10,3**–**28,5]	p < 0.001

### Bariatric surgery induces significant changes in GM

A total of 8.160.105 reads were obtained (median value = 78.519 reads) accounting for a total of 907 bacterial taxa. An additional taxonomic filter was applied to remove low-prevalence taxa (Patescibacteria and Tenericutes), generating a final dataset that accounted 8.159.323 reads (median value = 78.519 reads) accounting for 904 taxa. To minimize the effect of the sequencing depth variation across samples, normalization to reads median reads was performed.

**Fig. 2:**
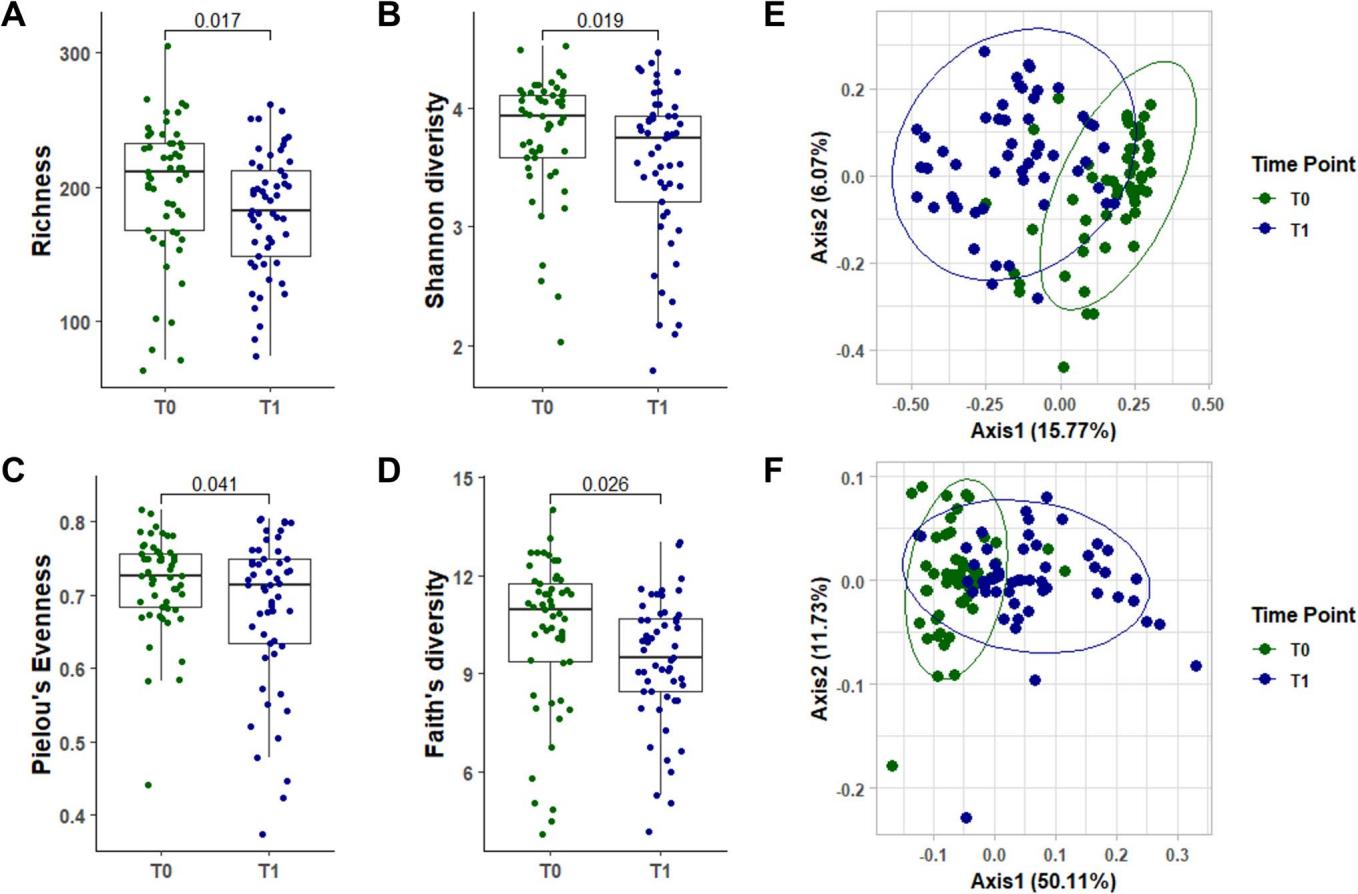
Bariatric surgery significantly impacts on gut microbiota alpha and beta diversity. Alpha diversity measured as richness (i.e. observed species measure) **A**, Shannon diversity index **B**, Pielou’s Evenness **C** and Faith’s diversity **D** was represented as dot plot chart (T0, showing samples pre-bariatric surgery, noted as dark green and T1, showing samples post-bariatric surgery noted as dark blue). Box plot chart reports median and 25th and 75th percentiles. Statistical differences were evaluated using Wilcoxon test for paired samples, followed by Bonferroni’s correction. All alpha diversity measures showed lower values post-intervention (T1) compared to the basal measurement. Beta diversity was investigated using *Weighted Unifrac* and *Bray–Curtis* distances and represented by Principal coordinates analysis (PCoA) (**E** and **F**, respectively). Statistical differences between the two groups were assessed by Permutational multivariate analysis of variance (PERMANOVA) test

Bacterial communities of samples at T0 significantly differed from those at T1 counterparts, when assessed either by the community richness (observed species, *p* = 0.017), the Shannon diversity index (*p* = 0.019), the community equitability (Pielou’s evenness, *p* = 0.041) or phylogenetic diversity (Faith’s diversity, *p* = 0.026) (Fig. 2a–d, Supplementary Table 2). Weighted Unifrac PCoA-based representation of the gut microbiota composition showed that samples from T0, though partially overlapping, were significantly different from those of T1 (*p* = 0.001, *R2* = 0.215) (Fig. 2e). These findings were confirmed by the Bray Curtis PCoA-based representation for the same samples (*p* = 0.001, *R2* = 0.092) (Fig. 2f).

**Fig. 3:**
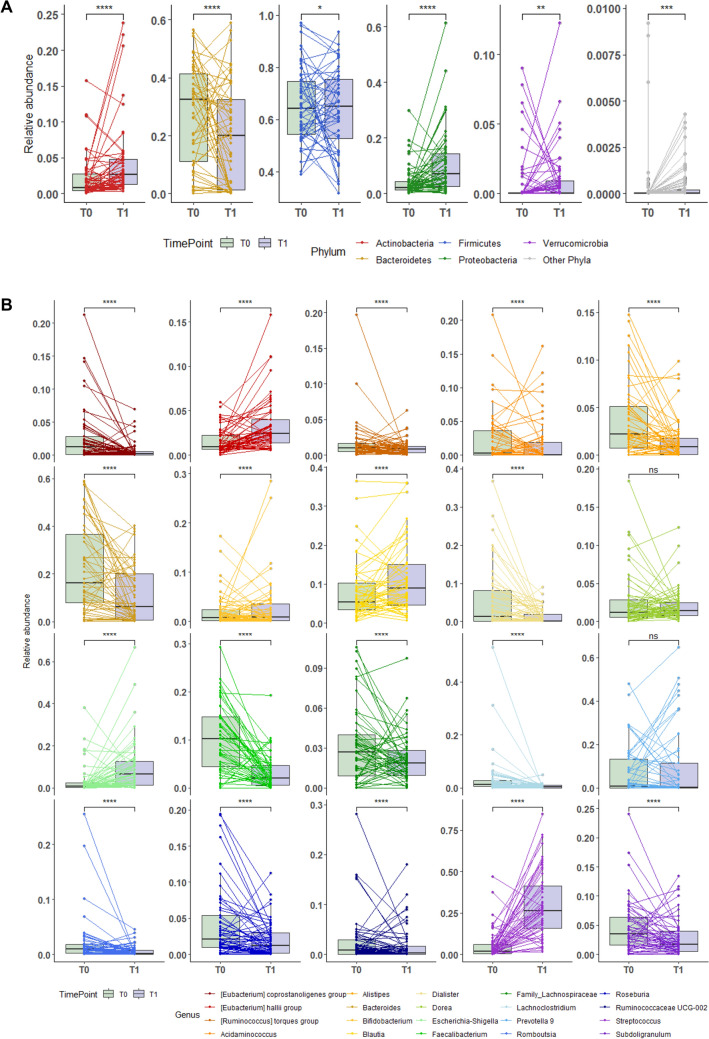
Bariatric surgery determines significant changes in the bacterial community. Taxonomic profile of the gut microbiota pre- and post-bariatric surgery (T0 and T1, respectively) was investigated at phylum (**A**) and genus level (**B**). Five main phyla (Actinobacteria, Bacteroidetes, Firmicutes, Proteobacteria and Verrucomicrobia) were detected, while Cyanobacteria and Fusobacteria (relative abundance less than 0.01%) were grouped as other phyla. Relative abundance at genus level was investigated on the 20 most representative genera. Each line links samples belonging to the same patient, whereas box plot chart is representative of the median and 25th and 75th percentiles. Statistical differences were inferred using Wilcoxon test for paired samples followed by Bonferroni’s correction. Supplementary Table 1 and supplementary table reports phylum and genus level relative abundance and statistical significance

Furthermore, relative abundance analysis of bacterial phylum (Supplementary Table 2) showed that Actinobacteria, Proteobacteria and Verrucomicrobia were more dominant (*p* < 0.001, *p* < 0.001 and *p* = 0.009, respectively) while Bacteroidetes and Firmicutes were less abundant in post-bariatric surgery samples (T1) than in baseline samples (T0) (*p* < 0.001 and *p* = 0.035, respectively) (Fig. 3a). Analysis at the genus level (Supplementary Table 3), indicates that Bacteroides and Faecalibacterium were more dominant at T0 (both showing *p* < 0.001), whereas Escherichia-Shigella and Streptococcus were the most abundant at T1 (both showing *p* < 0.001) (Fig. 3b).

**Fig. 4:**
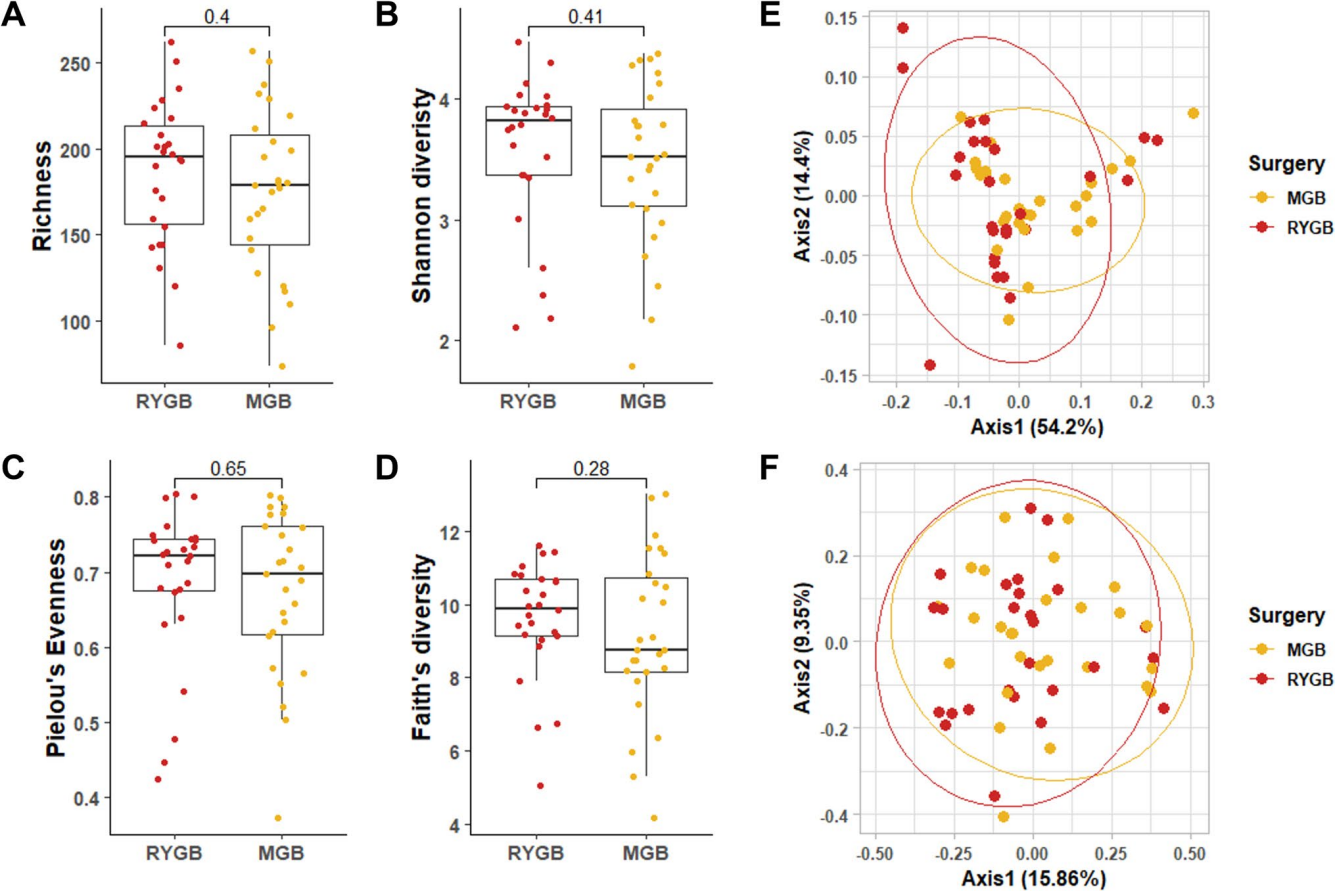
Bariatric surgery’s type does not impact on gut microbiota alpha and beta diversity. Gut microbiota of patients undergoing to Roux-en-Y Gastric Bypass (RYGB, red) or One Anastomosis/Mini Gastric Bypass (OAGB/MGB) interventions was analyzed. Alpha diversity was assessed as richness (i.e., observed species measure) (**A**), Shannon diversity index (**B**), Pielou’s Evenness (**C**) and Faith’s diversity (**D**). Statistical differences were evaluated using Kruskal–Wallis test. Alpha diversity did not show differences between RYGB and MGB. Principal coordinates analysis (PCoA) was used to represent Weighted Unifrac and Bray–Curtis beta diversity (**E** and **F**, respectively). Statistical differences between the two groups were assessed by Permutational multivariate analysis of variance (PERMANOVA)

**Fig. 5:**
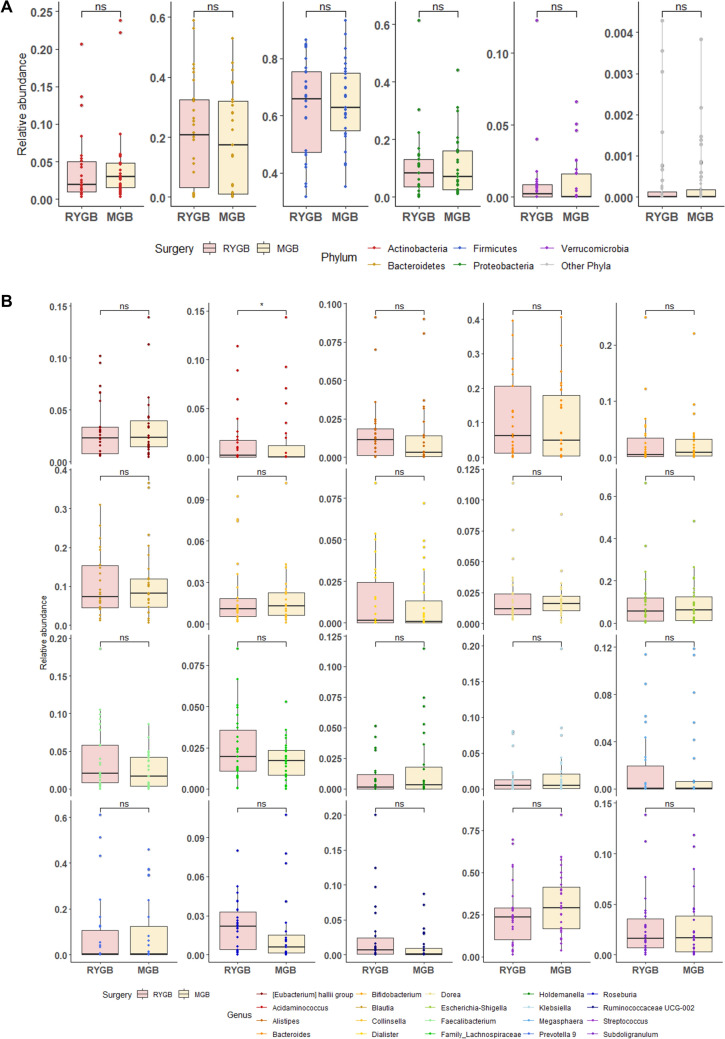
Microbial community composition changes following bariatric surgery. Taxonomic profile of the gut microbiota pf patients sorted by type bariatric surgery: Roux-en-Y Gastric Bypass (RYGB, red) or one Anastomosis/Mini Gastric Bypass (OAGB/MGB, yellow). Relative abundance at phylum (**A**) and genus (**B**) levels were reported. Supplementary Tables 4 and 5 describe phylum and genus level relative abundance and statistical significance

When investigating whether the two types of BMS can differentially impact on GM composition by measuring differences in α and β diversity at T1, α diversity indexes were not differentially affected depending on the RYGB or OAGB surgery, as represented in Fig. 4a–d and Supplementary Table 5. Alpha diversity was assessed as richness (i.e., observed species measure) (a), Shannon diversity index (b), Pielou’s Evenness (c) and Faith’s diversity (d), with no differences between the two bypass surgeries. Similarly, no differences were detected at Phylum and Genus level (Fig. 5, Supplementary Tables 6, 7). These results indicate that BMS promotes a change in GM composition, with no differences between the two types of bypass surgery performed. Statistical differences were evaluated using Kruskal–Wallis test.

### Firmicutes and Bacteroidetes ratio as a biomarker for bariatric surgery outcomes

**Fig. 6:**
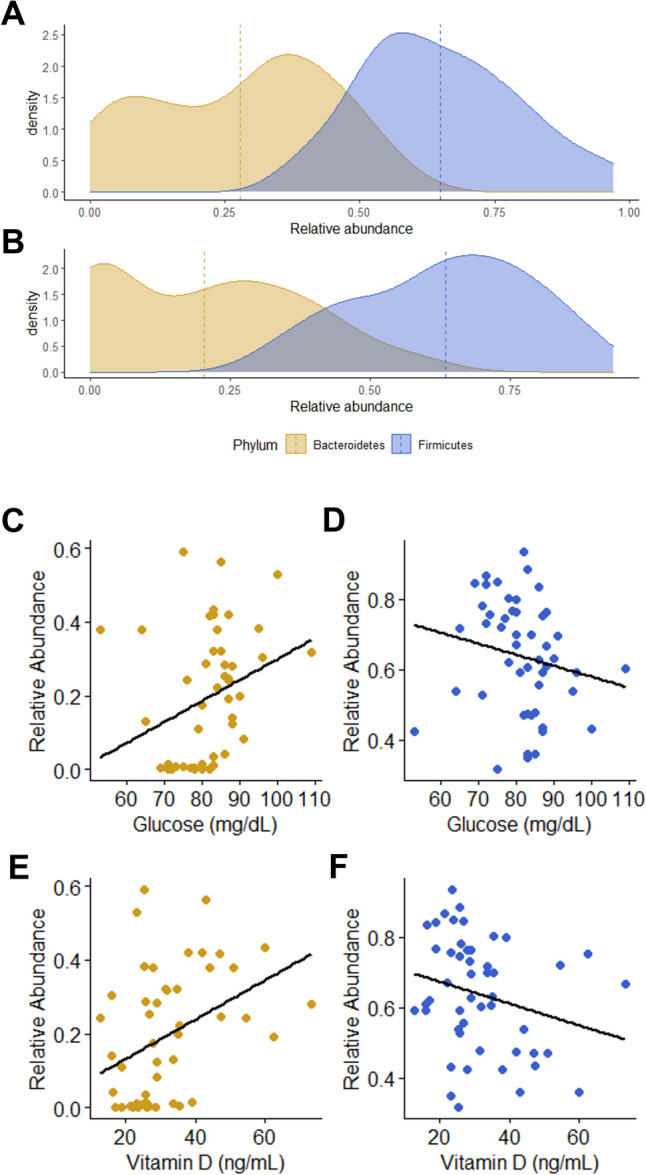
Firmicutes/Bacteroidetes ratio appears changed following bariatric surgery and their relative abundance is diversely related with serum levels of glucose and vitamin D. Firmicutes and Bacteroidetes relative abundances were selected and reported as density plot (dotted lines represent mean values) pre- and post-bariatric surgery intervention (**A** and **B**, respectively). Glucose and vitamin D serum level were corelated with relative abundances of Bacteroidetes (**C** and **E**, respectively) and Firmicutes (**D** and **F**, respectively) by Spearman’s rank correlation test

In our cohort, samples at T0 showed a F/B ratio median value of 1.98 [1.29–7.87], whereas at T1 the F/B ratio median value accounted a value of 3.15 [1.59–55.11] (Fig. 6a, b), mostly due to a decrease in Bacteroidetes abundance rather than an increase in Firmicutes abundance at T1.

We observed a statistically significant decrease of glucose and an increase of vitamin D levels following BMS (Table 1, Fig. 6c–f). When assessing the relationship between Firmicutes and Bacteroidetes relative abundances and the serum concentrations of these two parameters, Bacteroidetes relative abundance positively correlated with glucose (*p* = 0.002, rho = 0.437) and vitamin D (*p* = 0.002, rho = 0.427) serum levels, whereas Firmicutes relative abundance negatively correlated with glucose (*p* = 0.0395, rho = −0.295) and in part also with vitamin D (*p* = 0.072, rho = −0.259) serum levels.

## Discussion

To date the comparison between RYGB and OAGB remains elusive, due to the lack of worldwide accepted standard procedures [33–35]. This is the first prospective, multicenter, randomized study evaluating the GM shift in a very homogeneous cohort subjected to OAGB vs RYGB ≥ 24 months after surgery, reporting serum biomarkers measurements to assess metabolic and nutritional status. The high homogeneity in the patient’s selection and surgical protocols used (i.e., length of the limb, gastric pouch volume, anastomotic lengths) is a major strength of the study. No difference in total weight loss (TWL) and BMI one-year post-surgery was observed comparing the two study groups. As expected overall significant differences were found in both anthropometric and serum nutritional biomarkers when basal, preoperative, and 2 years later values were compared.

No previous study, at the best of our knowledge, investigated GM changes following RYGB and OAGB in a longitudinal cohort to explore the impact of the different bypass configurations.

A recent study depicts GM variations in patients following OAGB, highlighting a reduction in Shannon diversity and modifications in relative abundance at Phylum and Genus levels, whereas predominant Firmicutes and bacteria of the genus Eubacterium, Fusicatenibacter and Subdoligranulum decreased, Proteobacteria and Actinobacteria and bacteria of the Streptococcus genus increased [36]. Similarly, Izhak and colleagues describe a reduction of Firmicutes relative abundance followed by an increase in Proteobacteria, Fusobacteria, Bacteroidetes and Verrucomicrobia [37]. In line with these observations, a reduction in Firmicutes and a parallel increase in Bacteroidetes and Proteobacteria and of the Roseburia genus was observed following RYGB [38]. In our cohort, samples collected at T1 showed significant decrease in overall microbial diversity, in line with previous findings [39]. In contrast with previous reports, Firmicutes and Verrucomicrobia phyla did not significantly change, while Actinobacteria and Proteobacteria relative abundances increased compared to Bacteroidetes. These findings do not recapitulate previous observations, though it is important to highlight that we analyzed microbiota composition two year after surgery, in contrast with Kanial et al. that analyzed GM only after 6 months after surgery [36].

Our results confirm previous findings indicating a relative increase of Proteobacteria after bariatric surgery, which correlates with an improvement of metabolic functions and lowering of inflammatory parameters after RYGB, likely resulting from the increased abundance of anaerobic bacteria [38–40]. A characterization of the Proteobacteria colonizing the gut and specific anatomical niches, following BMS, may be important to: a) understand their functional role; b) identify the specific genes involved in the key biological processes; c) improve a generation of innovative probiotics based on these species. Functional GM characterization with metaproteomic studies may shed light on these issues.

Intriguingly, a correlation analysis between obese subjects and normal-weight people indicated that body fatness and waist circumference negatively correlated with Bacteroidetes taxa, while Firmicutes taxa positively correlated with body fat and negatively with muscle mass and/or physical activity level [38]. Data obtained from animal models revealed a significant increase of the Firmicutes and decrease of the Bacteroidetes relative abundances in obese mice and the F/B ratio is enhanced also in obese people [37]. The usefulness of the F/B ratio as a biomarker in BMS patients is controversial due to environmental influences, including diet and physical activity, and differences in surgical procedures or on postoperative period studied [21]. Several studies have described that the GM of obese humans exhibits a higher Firmicutes/Bacteroidetes (F/B) ratio compared with normal-weight individuals, suggesting the use of the F/B ratio as a biomarker [41–43]. We studied a homogeneous cohort also in terms of post-intervention supplements and multivitamins [40, 44] and we observed an increase in the F/B ratio two year post-surgery in comparison with basal samples, mostly due to a decrease in Bacteroidetes abundance rather than an increase in Firmicutes abundance. It is possible that Firmicutes may be not properly affected by bariatric surgery, but each nutritional modification may impact on Bacteroidetes phylum. These findings are not in agreement to what previously observed [38], mostly due to a decrease in Bacteroidetes abundance rather than an increase in Firmicutes abundance at T1.

Although the relationship between microbes and host metabolism may be bidirectional, we attempted also to identify possible biomarkers to evaluate the surgical outcome. Host glucose and specific bacteria can alter the intestinal barrier and impair immune functions and antimicrobial defenses [45, 46]. In contrast to previous observation, glucose serum levels at T1 positively correlated with Bacteroidetes and Firmicutes abundances [47]. Maintaining an optimal vitamin D status significantly induces change in Firmicutes, Bacteroidetes and Actinobacteria and may inhibit pro-inflammatory conditions [48]. Postoperative protocol included a strictly controlled diet and the same regime of vitamins and minerals supplementation. In this way, the GM modifications due to postoperative protocol, including previously mentioned factors were mitigated and constant in all enrolled patients. The primary endpoint of the study was to identify any differences between the GM modifications produced by the different bypass configuration, so we do not consider this topic as a study limitation.

To the best of our knowledge, this is the first prospective study exploring at baseline the different impact of obesity per se and the metabolic syndrome MS in a selected cohort of patients with severe obesity (BMI 40–55 kg/m^2^) candidate for bariatric/metabolic surgery. The preliminary results (at T0) published prior to this final report suggested that in the selected cohort, the pre-MS and MS conditions did not affect GM profile. Obesity per se seemed to be an independent determinant of GM profile changes. The preoperative assessment of patients, candidates for BMS could include GM ecology thereby, adding information to the ‘‘patient’s profile”. These data could be relevant for the follow-up and could influence the clinical practice.

Overall, analysis of the changes in the composition of microbial communities between T0 and T1 corroborates the observed differences in α-diversity and β-diversity in gut microbiota two year after bariatric surgery. Hence, although we did not detect marked differences in α-diversity between T0 and T1, BMS intervention prompted a change in microbiota composition that resulted in a decrease in the overall number of bacterial species and in a reduction in microbial phylogenetic diversity [49, 50]. So, we can presume that differences in GM composition between T0 and T1 depend upon changes in species presence and their relative abundance rather than an overall composition.

The main finding of this study is that BMS promotes significant changes in gut microbiota composition, with a reduction in alpha and beta diversity in samples collected at T1 (post-surgery) compared to samples collected before surgery (T0), and these changes are associated with a dramatic improvement in key clinical parameters. It follows that the changes in gut microbiota may potentially serve as a biomarker to monitor effectiveness of surgery, while it will be of interest to investigate whether in the medium-long-term that certain microbial signatures correlate with clinical features. Of course, since microbiota composition is affected by multiple parameters including but limited to diet, environment and other medical therapies, it will be important to determine the contribution of BMS to these changes. At the same time, BMS represents techniques to remodel the intestinal microbiome and, based of its different techniques, it does not completely follow the same phenotype because not all bariatric procedures have the same physiopathology. BMS procedures are categorized based on physiological changes and their effectiveness varies, depending on the type of procedure [51]. The effectiveness of the bariatric procedures is associated with their effects on the GM, as there are changes in the composition, gene content, and fermentation profiles of microbes in the gut. RYGB was shown to modify the GM significantly when compared to the most common performed bariatric procedure worldwide, the sleeve gastrectomy [52]. Up to date, there were no reports of the same metabolic benefits due to the postoperative GM modifications after OAGB. The prospective implications of this research in the clinical practice might be the increased use in the BMS techniques that modify the food flow on one hand, and on other hand, is shown that OAGB is as efficient as RYGB when speaking on GM modifications. Further studies should confirm these results and clinical benefits on long-term. Moreover, follow-up studies on this cohort may provide indications on the long-term outcomes of having a given microbiota signature. The emergence of slightly different GM profile postoperatively may be related to clinical condition or may play a key role in the long-term outcome and may represent a biomarker to follow bariatric surgery’s outcomes.

### Limitations

Most significant is that the study is probably underpowered to evaluate the primary outcome–changes in gut microbiome after RYGB vs OAGB. Reasons for 30% lost to follow-up include the covid pandemics that disrupted normal follow-up, as well as either lost to follow-up, or incomplete required 24-months follow-up. Still, the *sample size* (*n* = 54, 27 for group) was calculated fixing alpha error, power, *effect size* and standard deviation.

## Conclusion

BMS promotes a dramatic change in GM composition on medium term postoperatively. Our results demonstrated that RYGB and OAGB similarly affect GM composition at least 24 months after surgery. Thus, the different gastric bypass configuration does not induce different GM profile as possible driver of the metabolic effect and weigh loss.

## Supplementary Information

Below is the link to the electronic supplementary material.

Supplementary file1 (DOCX 29 kb)

## Data Availability

The metadata that support the findings of this study will be available through the dedicated online platform https://test-3mstudy.pantheonsite.io/user/login and will be available for secondary analysis once the study has been completed. All data generated or analyzed and included in this published article, and its supplementary information files, as well as previous report will be available. Data are however available from the authors upon reasonable request and with permission of further publication.
