# Supplementary material for: Short-term gut microbiota’s shift after laparoscopic Roux-en-Y vs one anastomosis gastric bypass: results of a multicenter randomized control trial
Source: Surg Endosc. 2024 Sep 18;38(11):6643–56. doi: 10.1007/s00464-024-11154-6 (PMC11525425; doi:10.1007/s00464-024-11154-6)
Supplement: Supplementary file 1 — Supplementary file1 (DOCX 29 kb) [file 464_2024_11154_MOESM1_ESM.docx]

**Supplementary Table 1.** Anthropometric and biochemical parameters of the patients participating to the study stratified following Roux-en-Y Gastric Bypass (RYGB) and One Anastomosis Gastric Bypass (OAGB). Results of Chi Square or Welch t-test for categorical or normally distributed numeric variables have been reported. Data were reported as median and 25^th^-75^th^ percentile.

|  | RYGB (n°=27)  median [Q1-Q3] | | OAGB (n°=27)  median [Q1-Q3] | | *Welch T-test* | |  |
| --- | --- | --- | --- | --- | --- | --- | --- |
| Sex (female) | | 18 | | 15 | | *p = 0.577** | |
| Height (cm) | 165,0 [157,0-176,0] | | 165,0 [160,0-177,0] | | *p = 0,907* | |  |
| Weight (kg) | 81,0 [72,0-88,4] | | 80,0 [74,0-94,0] | | *p = 0,975* | |  |
| BMI (kg/m^2^) | 29,54 [26,3-31,9] | | 29,7 [25,7-31,2] | | *p = 0,817* | |  |
| Neck circum (cm) | 36,0 [34,0-38,0] | | 36,0 [34,0-40,0] | | *p = 0,278* | |  |
| Waist (cm) | 99,0 [87,0-105,0] | | 94,0 [87,0-105,0] | | *p = 0,868* | |  |
| Hip (cm) | 107,0 [99,0-116,0] | | 110,0 [101,0-116,0] | | *p = 0,776* | |  |
| Albumin (g/dL) | 4,1 [3,9-4,3] | | 4,1 [3,9-4,3] | | *p = 0,262* | |  |
| Hemoglobin (g/dL) | 12,9 [12,0-13,8] | | 13,3 [12,7-14,2] | | *p = 0,256* | |  |
| HbA1c (mmol/ml) | 34,0 [29,0-36,0] | | 34,0 [30,0-38,0] | | *p = 0,436* | |  |
| Glucose (mg/dL) | 83,0 [75,0-87,0] | | 83,0 [73,0-88,0] | | *p = 0,738* | |  |
| Triglycerides (mg/dL) | 80,0 [65,0-103,0] | | 76,0 [63,0-105,0] | | *p = 0,832* | |  |
| Cholesterol (mg/dL) | 150,0 [126,0-171,0] | | 155,0 [138,0-180,0] | | *p = 0,279* | |  |
| HDL (mg/dL) | 48,0 [35,0-58,0] | | 56,0 [40,0-61,0] | | *p = 0,213* | |  |
| LDL (mg/dL) | 89,0 [70,0-103,0] | | 92,4 [77,0-122,0] | | *p = 0,351* | |  |
| Ferritin (ng/mL) | 50,0 [15,0-126,0] | | 36,8 [17,0-82,0] | | *p = 0,934* | |  |
| Iron (μg/dL) | 75,0 [55,0-90,7] | | 80,0 [55,0-102,0] | | *p = 0,518* | |  |
| Calcium (mg/dL) | 9,4 [9,0-9,7] | | 9,2 [8,9-9,5] | | *p = 0,496* | |  |
| Vitamin D (ng/mL) | 29,0 [23,3-43,0] | | 27,9 [23,5-34,9] | | *p = 0,587* | |  |
| Vitamin B12 (pg/mL) | 348,0 [301,0-477,0] | | 394,0 [289,0-535,0] | | *p = 0,632* | |  |
| Cortisol (μg/mL) | 15,2 [11,7-66,0] | | 12,0 [8,9-22,1] | | *p = 0,143* | |  |

**Supplementary table 2:** Alpha diversity values at baseline and post-surgery. Data were reported as median and 25^th^-75^th^ percentile. Wilcoxon statistical test for paired data was used to assess statistical significance.

| **Alpha diversity** | **T0**  **median [Q1-Q3]** | **T1**  **median [Q1-Q3]** | ***Wilcoxon*** |
| --- | --- | --- | --- |
| **Richness** | 211,0 [168,0-233,0] | 182,0 [148,0-212,0] | ***p = 0,017*** |
| **Shannon diversity** | 3,93 [3,58-4,11] | 3,74 [3,21-3,93] | ***p = 0,019*** |
| **Pielou’s evenness** | 0,72 [0,68-0,75] | 0,70 [0,63-0,74] | ***p = 0,041*** |
| **Faith’s diversity** | 10,94 [9,36-11,73] | 9,48 [8,46-10,69] | ***p = 0,026*** |

**Supplementary table 3:** Relative abundances of the five main phyla detected at the basal time point and 24 months post-bariatric surgery. Data were reported as mean, median and 25^th^-75^th^ percentile. Wilcoxon statistical test for paired data was used to assess statistical significance.

| **Phylum** | **T0**  **Mean; median [Q1-Q3]** | **T1**  **Mean; median [Q1-Q3]** | ***Wilcoxon*** |
| --- | --- | --- | --- |
| **Actinobacteria** | 0,027; 0,021 [0,004-0,027] | 0,043; 0,026 [0,013-0,048] | ***p < 0,001*** |
| **Bacteroidetes** | 0,277; 0,324 [0,112-0,415] | 0,204; 0,200 [0,013-0,324] | ***p < 0,001*** |
| **Firmicutes** | 0,650; 0,640 [0,543-0,746] | 0,634; 0,650 [0,527-0,754] | ***p = 0,035*** |
| **Proteobacteria** | 0,042; 0,020 [0,0140-0,043] | 0,108; 0,070 [0,027-0,144] | ***p < 0,001*** |
| **Verrucomicrobia** | 0,008; 0,000 [0,000-0,0006] | 0,010; 0,0002 [0,000-0,009] | ***p = 0,009*** |
| **Other Phyla** | 0,0003; 0,000 [0,000-0,000] | 0,0003; 0,000 [0,000-0,0002] | ***p < 0,001*** |

**Supplementary table 4:** Relative abundances of the top 20 genera detected at the basal time point and 24 months post-bariatric surgery. Data were reported as mean, median and 25^th^-75^th^ percentile. *Wilcoxon* statistical test for paired data was used to assess statistical significance.

| **Genus** | **T0**  **Mean; median [Q1-Q3]** | **T1**  **Mean; median [Q1-Q3]** | ***Wilcoxon*** |
| --- | --- | --- | --- |
| **[Eubacterium] coprostanoligenes group** | 0,028; 0,012 [0,001-0,028] | 0,007; 0,002 [0,001-0,006] | ***p < 0,001*** |
| **[Eubacterium] hallii group** | 0,015; 0,010 [0,006-0,022] | 0,033; 0,024 [0,014-0,040] | ***p < 0,001*** |
| **[Ruminococcus] torques group** | 0,017; 0,010 [0,005-0,016] | 0,011; 0,008 [0,003-0,016] | ***p < 0,001*** |
| **Acidaminococcus** | 0,023; 0,002 [0,000-0,037] | 0,000; 0,000 [0,000-0,018] | ***p < 0,001*** |
| **Alistipes** | 0,037; 0,022 [0,007-0,051] | 0,015; 0,009 [0,000-0,018] | ***p < 0,001*** |
| **Bacteroides** | 0,226; 0,161 [0,080-0,365] | 0,111; 0,061 [0,005-0,202] | ***p < 0,001*** |
| **Bifidobacterium** | 0,021; 0,008 [0,001-0,025] | 0,029; 0,008 [0,001-0,035] | ***p < 0,001*** |
| **Blautia** | 0,081; 0,053 [0,034-0,104] | 0,111; 0,090 [0,046-0,151] | ***p < 0,001*** |
| **Dialister** | 0,056; 0,013 [0,000-0,081] | 0,013; 0,001 [0,000-0,019] | ***p < 0,001*** |
| **Dorea** | 0,027; 0,012 [0,006-0,029] | 0,021; 0,014 [0,008-0,025] | *p = 0,68* |
| **Escherichia-Shigella** | 0,033; 0,007 [0,002-0,023] | 0,098; 0,065 [0,013-0,126] | ***p < 0,001*** |
| **Faecalibacterium** | 0,102; 0,102 [0,045-0,148] | 0,033; 0,020 [0,006-0,047] | ***p < 0,001*** |
| **Family_Lachnospiraceae** | 0,032; 0,027 [0,009-0,040] | 0,022; 0,019 [0,010-0,028] | ***p < 0,001*** |
| **Lachnoclostridium** | 0,037; 0,012 [0,006-0,028] | 0,006; 0,004 [0,000-0,010] | ***p < 0,001*** |
| **Prevotella 9** | 0,073; 0,007 [0,000-0,132] | 0,096; 0,002 [0,000-0,114] | *p = 0,23* |
| **Romboutsia** | 0,022; 0,009 [0,003-0,018] | 0,006; 0,002 [0,000-0,008] | ***p < 0,001*** |
| **Roseburia** | 0,042; 0,021 [0,010-0,054] | 0,020; 0,013 [0,002-0,030] | ***p < 0,001*** |
| **Ruminococcaceae UCG-002** | 0,026; 0,008 [0,001-0,029] | 0,019; 0,003 [0,000-0,016] | ***p < 0,001*** |
| **Streptococcus** | 0,055; 0,020 [0,005-0,061] | 0,303; 0,264 [0,158-0,412] | ***p < 0,001*** |
| **Subdoligranulum** | 0,047; 0,035 [0,016-0,064] | 0,028; 0,017 [0,005-0,040] | ***p < 0,001*** |

**Supplementary table 5:** Alpha diversity values in patients undergoing RYGB and OAGB. Data were reported as median and 25^th^-75^th^ percentile. Wilcoxon statistical test for paired data was used to assess statistical significance.

| **Alpha diversity** | **OAGB**  **median [Q1-Q3]** | **RYGB**  **median [Q1-Q3]** | ***Wilcoxon*** |
| --- | --- | --- | --- |
| **Richness** | 179,0 [144,5-208,0] | 195,5 [156,0-213,5] | *p = 0,400* |
| **Shannon diversity** | 3,52 [3,11-3,91] | 3,82 [3,37-3,94] | *p = 0,410* |
| **Pielou’s evenness** | 0,70 [0,62-0,76] | 0,72 [0,64-0,74] | *p = 0,650* |
| **Faith’s diversity** | 8,76 [8,17-10,73] | 9,90 [9,14-10,69] | *p = 0,280* |

**Supplementary table 6:** Relative abundances of the five main phyla detected in stool samples of patients stratified based on Roux-en-Y Gastric Bypass (RYGB) and One Anastomosis Gastric Bypass (OAGB) 24 months post-bariatric surgery. Data were reported as mean, median and 25^th^-75^th^ percentile. Wilcoxon statistical test for paired data was used to assess statistical significance.

| **Phylum** | **OAGB**  **Mean; median [Q1-Q3]** | **RYGB**  **Mean; median [Q1-Q3]** | ***Wilcoxon*** |
| --- | --- | --- | --- |
| **Actinobacteria** | 0,046; 0,030 [0,016-0,048] | 0,040; 0,019 [0,010-0,050] | *p = 0,330* |
| **Bacteroidetes** | 0,189; 0,175 [0,010-0,321] | 0,219; 0,207 [0,031-0,326] | *p = 0,530* |
| **Firmicutes** | 0,647; 0,628 [0,547-0,750] | 0,622; 0,659 [0,473-0,754] | *p = 0,720* |
| **Proteobacteria** | 0,107; 0,070 [0,025-0,159] | 0,109; 0,083 [0,036-0,131] | *p = 0,940* |
| **Verrucomicrobia** | 0,010; 0,000 [0,000-0,016] | 0,010; 0,002 [0,000-0,009] | *p = 0,950* |
| **Other Phyla** | 0,0003; 0,000 [0,000-0,0002] | 0,0003; 0,000 [0,000-0,0001] | *p = 0,360* |

**Supplementary table 7.** Relative abundances of the top 20 genera detected in stool samples of patients stratified based on Roux-en-Y Gastric Bypass (RYGB) and One Anastomosis Gastric Bypass (OAGB) 24 months post-bariatric surgery. Data were reported as mean, median and 25^th^-75^th^ percentile. Wilcoxon statistical test for paired data was used to assess statistical significance.

| **Genus** | **OAGB**  **Mean; median [Q1-Q3]** | **RYGB**  **Mean; median [Q1-Q3]** | ***Wilcoxon*** |
| --- | --- | --- | --- |
| **[Eubacterium] hallii group** | 0,032; 0,023 [0,014-0,039] | 0,031; 0,023 [0,008-0,033] | *p = 0,704* |
| **Acidaminococcus** | 0,016; 0,00 [0,00-0,012] | 0,016; 0,002 [0,0002-0,017] | *p = 0,043* |
| **Alistipes** | 0,014; 0,003 [0,0003-0,014] | 0,016; 0,011 [0,001-0,018] | *p = 0,259* |
| **Bacteroides** | 0,100; 0,048 [0,003-0,179] | 0,113; 0,062 [0,012-0,206] | *p = 0,566* |
| **Bifidobacterium** | 0,025; 0,008 [0,002-0,032] | 0,028; 0,004 [0,001-0,034] | *p = 0,590* |
| **Blautia** | 0,106; 0,073 [0,046-0,119] | 0,106; 0,074 [0,045-0,153] | *p = 0,979* |
| **Collinsella** | 0,018; 0,013 [0,006-0,013] | 0,019; 0,010 [0,005-0,018] | *p = 0,554* |
| **Dialister** | 0,011; 0,0008 [0,000-0,013] | 0,014; 0,002 [0,000-0,022] | *p = 0,701* |
| **Dorea** | 0,018; 0,016 [0,010-0,022] | 0,021; 0,012 [0,007-0,024] | *p = 0,744* |
| **Escherichia-Shigella** | 0,090; 0,062 [0,013-0,125] | 0,100; 0,055 [0,009-0,119] | *p = 0,757* |
| **Faecalibacterium** | 0,024; 0,016 [0,003-0,042] | 0,040; 0,020 [0,008-0,058] | *p = 0,230* |
| **Family_Lachnospiraceae** | 0,017; 0,017 [0,008-0,023] | 0,025; 0,019 [0,010-0,035] | *p = 0,363* |
| **Holdemanella** | 0,017; 0,003 [0,000-0,018] | 0,010; 0,001 [0,000-0,012] | *p = 0,475* |
| **Klebsiella** | 0,021; 0,004 [0,0006-0,020] | 0,016; 0,004 [0,0002-0,012] | *p = 0,707* |
| **Megasphaera** | 0,016; 0,0002 [0,000-0,006] | 0,017; 0,0003 [0,000-0,020] | *p = 0,526* |
| **Prevotella 9** | 0,093; 0,002 [0,000-0,122] | 0,090; 0,003 [0,0006-0,105] | *p = 0,809* |
| **Roseburia** | 0,017; 0,006 [0,001-0,015] | 0,023; 0,021 [0,004-0,033] | *p = 0,071* |
| **Ruminococcaceae UCG-002** | 0,011; 0,0006 [0,000-0,009] | 0,019; 0,003 [0,000-0,016] | *p = 0,052* |
| **Streptococcus** | 0,318; 0,291 [0,168-0,413] | 0,256; 0,234 [0,102-0,289] | *p = 0,138* |
| **Subdoligranulum** | 0,027; 0,016 [0,003-0,038] | 0,028; 0,016 [0,007-0,036] | *p = 0,702* |
